# Results of Re-vaccination in the Deaf and Dumb Institution of Paris

**Published:** 1841-08

**Authors:** 


*Results of Re-vaccination in the Deaf and Dumb Institution of Paris.*—The number re-vaccinated was 128—124 pupils, whose ages varied from ten to eighteen years, and four adults, servants of the institution. Of the entire number, 60 were males and 68 were females. The operation was performed from arm to arm; the vaccine lymph was abundant; and the number of punctures made in each arm varied from two to six.

In 25 of the individuals, there neither were any traces of previous vaccination on their arms, although they had no doubt been vaccinated in infancy, nor were there any marks of small pox on their face or body. (The mere absence, however, of cicatrices cannot be taken as a proof that the parties had never been vaccinated, nor had passed through variola.) Of these 25 cases, the vaccination produced no vesicles in 18; imperfect or false vesicles in four; and genuine cow-pox vesicles in three only.

Of seven individuals, who had distinct marks of small pox on their faces, limbs and bodies, the operation succeeded perfectly in two, and failed altogether in five of them.

In the remaining 94 cases, there was distinct cicatrices of a former vaccination, the number of these varying from one to four or six; in some, one or more cicatrices were observed on each arm, in others on one arm only.

Now of these 94 persons, ten exhibited distinct cow pox vesicles (after the re-vaccination,) 16 imperfect or bastard vesicles, and, in the remaining 70, the operation failed in producing any effects.

If we take, therefore, the entire number of persons "all well re-vaccinated by me," says M. Meniere, the reporter, "we find that in 15 cases only out of 128, regular cow-pox vesicles were formed over the punctures on the arms; in 20 the vesicles were imperfect or bastard; and in 93 none at all were developed. From these data it appears that the operation took effect in about the *one-eighth* of the whole; in about the same proportion, *one-eighth*, in those who had never had small pox, and who exhibited no traces of vaccine cicatrices on their arms, although they had been vaccinated at some former veriod of life; in *one-third* of those who had had small pox in their youth; and in about *one-tenth* of those in whom the cicatrices of a former vaccination were still distinct.

In estimating these results, it may be proper to attend to certain circumstances connected with the cases.

Of the fifteen persons in whom the re-vaccination took complete effect, ten were under thirteen years of age, and the other five were a few years older. In the two young girls, in whom it succeeded after previous small pox, (which had left numerous and most distinct traces on the face and elsewhere) five years had elapsed in one case, and seven in the other, since the date of the attack. Among the pupils who had been vaccinated in their infancy, and in whom the re-vaccination took complete effect, two were twelve years, and the third was fourteen years old.

From these data, we may infer that the preservative or counteracting power of small pox does not exceed that of cow-pox; since, under very similar circumstances, those who had passed through the two diseases were submitted to the same contagious influence, and experienced nearly the same results.

But we are unwilling to draw any general conclusions from the preceding report; as we are well aware that experiments must be made on a much more extensive scale before we can, safely do so.

In conclusion, we may state, that several infants were vaccinated for the first time from the vesicles on the arms of those in whom the second operation took effect and that the virus thus obtained seemed to be perfectly genuine and active.—*Med. Chirurg. Rev.* from *Journal des Connaiss. Med. Chirurg.* Sept. 1840.

